# Use of Platelet Rich Plasma Gel on Wound Healing: A Systematic Review and Meta-Analysis

**Published:** 2011-09-15

**Authors:** Marissa J. Carter, Carelyn P. Fylling, Laura K. S. Parnell

**Affiliations:** ^a^Strategic Solutions, Inc, Cody, WY; ^b^Cytomedix, Inc, Gaithersburg, MD; ^c^Precision Consulting, Missouri City, TX

## Abstract

**Objective:** Autologous platelet rich plasma is an advanced wound therapy used in hard-to-heal acute and chronic wounds. To better understand the use and clinical outcomes of the therapy, a systematic review of the published literature in cutaneous wounds was performed. **Methods:** Electronic and hand searches for randomized controlled trials and comparative group studies using platelet rich plasma therapy in cutaneous wounds and published over the last 10 years was conducted. Eligible studies compared the treatment to standard care or other interventions. All citations were screened and eligible studies were assessed for validity, quality, and bias using accepted scoring methods. The primary outcomes were effect of platelet rich plasma and control wound care on wound healing and related healing measurements. Secondary outcomes related to healing such as infection, pain, exudate, adverse events, and quality of life were also considered. The meta-analysis utilized appropriate statistical methods to determine the overall treatment effect on chronic and acute wound healing and infection. **Results:** The search terms resulted in 8577 citations and after removing duplicates and screening for protocol eligibility, a total of 24 papers were used. The meta-analysis of chronic wound studies revealed platelet rich plasma therapy is significantly favored for complete healing. The meta-analysis of acute wounds with primary closure studies demonstrated that presence of infection was reduced in platelet rich plasma treated wounds. **Conclusions:** This systematic review and meta-analysis of platelet rich plasma therapy in cutaneous wounds showed complete and partial wound healing was improved compared to control wound care.

Wound healing is a complex and dynamic process.^1^ Once a wound begins healing, normally the process resolves with complete wound closure. However, healing of acute and chronic wounds can become impaired by patient factors (ie, comorbidities) and/or wound factors (ie, infection).^2^ Restarting a wound with impaired healing is difficult because good standard wound care does not always provide an improved healing outcome and often more advanced therapies are employed.^3^^-^4

Platelet rich plasma (PRP) gel is considered to be advanced wound therapy for chronic and acute wounds. For more than 20 years, PRP gel has been used to stimulate wound healing. Autologous PRP gel consists of cytokines, growth factors, chemokines, and a fibrin scaffold derived from a patient's blood.^5^^-^6 The mechanism of action for PRP gel is thought to be the molecular and cellular induction of normal wound healing responses similar to that seen with platelet activation.^6^

Various studies evaluating PRP gel have been published over the years. Study design, study populations, clinical outcomes, and methodological quality vary widely between citations making concrete conclusions difficult. Currently, there are 3 systematic reviews on PRP therapy.^7^^-^9 One systematic review looked at tissue regeneration in randomized controlled trials (RCTs) in maxillofacial surgery, chronic ulcers, and surgical wounds.^7^ Another systematic review assessed healing of RCT diabetic ulcer studies.^8^ A third systematic review was a poster presentation on the results of a systematic review of healing chronic leg ulcers.^9^

The systematic review described herein was performed to specifically assess studies in cutaneous skin wounds treated with autologous PRP gel and standard wound care (control groups). Studies assessing healing information such as complete or partial wound healing, time to heal, healing trajectory, velocity or rate, and wound size reduction were considered critical for this systematic review.

## METHODS

### Selection criteria

For assessment of PRP treatment human intervention trials, only RCTs and comparative studies (ie, treatment/intervention groups compared with controls, or a group with run-in and comparable treatment data) published in peer-reviewed journals (articles, brief articles, case studies, or letters) or presented at scientific meetings (abstracts) were considered. Literature published between March 2001 and March 2011 was reviewed. Studies were eligible if the participants had a cutaneous ulcer or wound (including dehisced wounds, open surgical wounds, acute, or chronic wounds) that were treated with activation-processed PRP. Studies of patients with mixed origin wounds, subsets of different wound types, surgical wounds treated with PRP prior to closure and open, surgical wounds treated with PRP for secondary closure were included. In addition, inclusion eligibility required PRP studies to have a control treatment group (ie, placebo, wound care treatment). Noninferiority trials in which 2 types of PRP treatment were compared were also eligible. Studies in which the experimental group received other treatments were eligible provided that the control group also received the same treatment or care so that confounding was avoided and the systematic difference between the groups was only the primary intervention.

Studies focusing on burns, dental or jaw treatment, bone fractures, orthopedic injection, or plastic surgery were excluded because of the different healing characteristics of these wounds. Studies which used homologous/allogenic PRP procedures, lysates, freezing, or freeze-dried techniques to produce PRP, or were considered to be “fibrin glue,” were also excluded.

### Outcome measures

Eligible studies had to report at least one wound-healing parameter as an outcome measure, and/or associated parameters such as infection rates and incidence, pain measures, exudation management, quality-of-life measures, or net health benefits. Examples of wound-healing parameters included complete wound healing (proportions in each group or percentages provided N for each group was reported; Kaplan-Meier and Cox regressions); wound area reduction (mean or median, relative, absolute, or percentage); wound depth or volume reduction (same parameters as for area); healing rate (change in area or wound dimension per unit of time expressed in absolute terms or as a percentage), time to heal (mean or median, expressed in days or weeks), or comparison of clinical significant healing events, such as reaching a reduction of 50% or more in area using Kaplan-Meier or Cox regression. Outcomes could be unadjusted or adjusted for other covariates and factors, and compare baseline and final outcomes, or repeated measures. Follow-up for treatment/intervention trials had to be a minimum of 2 weeks.

### Search strategy

The Cochrane Library, Scopus, CINAHL, and PubMed databases were searched using combinations of the following terms: platelet rich plasma, platelet rich plasma gel(s), PRP, PRP gel(s), platelet gel(s), autologous growth factors, wound(s), chronic wound(s), chronic nonhealing wound(s), open, cutaneous wound(s), dehiscence, dehisced, surgical wound(s), diabetic ulcer, venous ulcer, pressure ulcer, sternal wound(s).

The journals *Wounds*, *Worldwide Wounds* and the clinical trial database clinicaltrials.gov were hand searched using the same terms. Identified reviews were also searched for additional references to RCTs and comparative studies not previously captured. Narrative reviews and editorials were examined for references of potential trials. Several experts in the field were also consulted for their knowledge of RCTs. After the initial selection of study abstracts appeared to meet selection criteria, 2 reviewers (M.J.C. and C.P.F.) evaluated each study in full to determine whether the study met the selection criteria and outcome measures.

Numbers of citations for each search term entered (or combination of search terms), numbers of papers fully examined, numbers of papers eligible for review, and numbers of papers excluded with reasons were recorded. Duplicates were removed after confirming identical publication information. Papers suspected of containing the same results published elsewhere were reviewed to determine the originality of the results and which paper best met the selection criteria outlined earlier.

### Initial quality assessment

The quality of each study was assessed using a method reported by Downs and Black^10^ and modified by Carter et al.^11^ The scoring sheet comprised 5 sections: reporting (quality of how the study data were reported), external validity (the generalizability of the study), internal validity (assessment of the potential for bias), internal validity (assessment of potential confounders that may compromise the study), and power (assessment of the power of the study to discriminate the effect sizes of the outcomes). Modifications of the original method included replacement of the external validity module with an approach based on the number of patients who would likely have been excluded from the study.^12^ The section is scored according to the category of the study—satisfactory: 3 points; problematic: 2 points; unsatisfactory: 1 point. The power module was truncated with the following scoring scheme: reported sample size calculation (for RCTs): 1 point; reported more than 1 calculation: 2 points; no reporting of sample size calculation: 0 points; power reported for at least 1 clinically important effect (for comparative studies): 1 point; reported for all clinically important effect: 2 points; no reporting of power for clinically important effects: 0 points. The total score possible was 29 points.

Bias was summarily reported using the SIGN grade methodology, which is defined as follows: ++ applies if all or most criteria from the checklist are fulfilled or where criteria are not fulfilled, the conclusions of the study or review are thought very unlikely to alter; + applies if some of the criteria from the checklist are fulfilled or where criteria are not fulfilled or are not adequately described, the conclusions of the study or review are thought unlikely to alter; – applies if few or no criteria from the checklist are fulfilled or where criteria are not fulfilled or are not adequately described, and the conclusions of the study or review are thought likely or very likely to alter.^13^ The grade assignment of study bias was accomplished by taking the total score of the external validity and internal validity (bias and confounding) sections of the quality assessment, and scoring as follows: 0 to 8 points (−); 9 to 12 points (+); 13 to 16 points (++). Scoring was carried out independently by M.J.C. and C.P.F. who then reconciled any discrepancies in subsequent discussion. Final grade assignments took into consideration serious flaws or inconsistencies, or other attributes that could decrease or increase initial grade assessment.^14^

### Data extraction and analysis

Outcomes were categorized by type, and for each one, the pretreatment and posttreatment numbers, median, or mean values (SD) were extracted where possible. To ensure that correct numbers were obtained, this process was performed by M.J.C. and checked by C.P.F. No investigators were contacted for further clarification. The number needed to treat was calculated for studies reporting complete wound healing, and where protocol analyses were used, the data were updated to reflect an intent-to-treat analysis. Data were imported into software (Revman 5.0 Information Management, Nordic Cochrane Centre, Copenhagen, Denmark) to calculate 95% confidence intervals and *P* values using fixed-effect models where possible. The Mantel-Haenszel method was used with risk difference as the effect measure in the case of dichotomous events, and the inverse variance method was used with the weighted mean difference (WMD) as the effect measure in the case of continuous (interval) data.

### Grading

After data extraction and initial quality assessment were complete, important and critical outcomes were agreed upon using consensus and quality assessment. Summary of findings for studies comparing use of PRP treatments against standard care were assessed using the GRADE system for each type of wound.^14^^-^16

### Meta-analysis

Meta-analysis (statistical pooling) was carried out on those studies that had the following compatible outcomes and reasonable clinical homogeneity: (1) complete wound healing; (2) superficial infection; and (3) pain reduction. Results from RCTs were pooled separately from other comparative studies. For dichotomous events, a fixed-effect model was employed that used the Mantel-Haenszel method with risk difference as the effect measure for easier interpretation. In the case of continuous (interval) data, a fixed-effect model was also employed using the inverse variance method with the effect measure of WMD (weighted mean difference). Statistical heterogeneity was assessed using the I^2^ (inconsistency) statistic, which indicates the percentage variation between studies that is a result of heterogeneity rather than chance.^17^ If the I^2^ (inconsistency) value was 30% or higher, meta-analysis was also conducted using a random effects model.

## RESULTS

### Study selection

**Figure 1:**
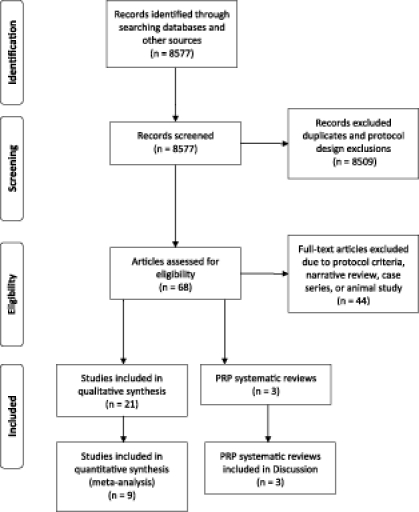
Systematic review of platelet rich plasma literature, March 2001 to March 2011. Template from Moher D, Liberati A, Tetzlaff J, Altman DG; The PRISMA Group (2009). Preferred Reporting Items for Systematic Reviews and Meta-Analyses: The PRISMA Statement. *PLoS Med*. 6(6): e1000097. doi:10.1371/journal.pmed1000097

The protocol search terms (see “Methods”) resulted in 8577 citations, the majority of which were duplicates (Fig 1). Potentially eligible studies (n = 68) were identified per protocol criteria.^18^^-^82 There were 44 papers excluded for one or more of the following reasons: narrative review, noncomparative study and/or case series, confounded with other treatments, cost-effectiveness study, insufficient outcome data reported, noncutaneous wounds, generic wound care research paper, reported outcomes did not include protocol eligibility criteria, non-PRP systematic review, freeze-dried PRP, allogenic PRP, frozen platelets, letter to editor discussing ineligible RCT, survey, and animal study.^39^^-^82

Twenty-one studies on the subject were identified and used (Fig 1).^18^^-^38 In addition, 3 systematic reviews on PRP use were found and detailed in the “Discussion” section.^7^^-^9 The reviewers agreed on 95.8% of the quality analysis items scored with a kappa of 0.899. Eligible PRP publications consisted of 12 RCTs, 3 cohort studies, 5 comparative study designs, and 1 retrospective analysis with propensity scoring. Table 1 describes the study design, enrolled subjects, wounds, and wound care treatments of each. Within the eligible studies, 3 main types of wounds which were treated with PRP were identified: (1) open, chronic wounds,^18^^-^24 (2) acute surgical wounds with primary closure,^25^^-^35 and (3) acute surgical wounds with secondary closure.^36^^-^38

### Quality analysis

Three citations with the least bias were RCTs in primary closure acute wounds^25^^,^^28^^,^^30^ (Table 2). Study quality varied greatly between papers. Chronic wound studies had 2 RCTs score − and 3 comparative studies score +. Studies in acute wounds with primary closure had 3 RCTs score ++, 3 score − and 5 comparative studies score +. Acute wounds with secondary closure studies had 2 RCTs score + and 1 comparative study score − (Table 2).

### Study outcomes

Outcomes associated with wound healing directly (ie, healing, size reduction) or indirectly (healing impairments, complications) were analyzed. These outcomes were judged to be critical or important since time to heal, complications (ie, amputations or life-threatening situations), and quality of life (QoL) can significantly impact wound healing. Data analyses for clinical outcomes for each article are in Tables 3 to 5.

#### Chronic wounds

Of 4 RCTs, 2 were statistically significant for complete wound healing compared to saline gel or no topical treatment.^19^^-^20 It should be noted that the 2 studies that did not detect significance at weeks 5 and 8 did have statistically significant wound area reductions compared to saline gauze or no topical treatment suggesting that a longer study period would have detected complete healing^18^^,^^21^ (Table 3). A non-RCT, comparative designed study showed consistently higher significant relative risks for complete wound healing in favor of platelet releasate compared to no topical treatment based on 26,599 subjects^23^ (Table 3). Two RCT studies evaluated time to heal and both showed significant improvements in PRP-treated subjects versus saline gel or no topical treatment^19^^-^20 (Table 3). Platelet rich plasma subjects in a historical cohort study required significantly fewer days to complete healing compared to hyaluronic acid–dressed wounds^24^ (Tables 3 and 4). Two RCT studies showed statistically significant differences in wound area reduction compared to saline gauze or no topical treatment controls.^18^^,^^21^ Similarly, a non-RCT, comparative study showed significant area and depth reductions with 2.5- to 3.5-fold decrease in time to reach 50% compared to pretreatment moist wound care controls.^22^ Adverse events were consistently lower for PRP groups than for controls of saline gauze, saline gel or no topical treatment.^18^^-^20

#### Acute primary closure wounds

Only 2 RCT and 2 non-RCT comparative studies specifically looked at wound-healing outcomes (Tables 3 and 5).^26^^,^^28^^,^^31^^,^^34^ One RCT detected a statistically significant difference in complete wound healing for PRP compared to no topical treatment during a short 2-week follow-up^28^ (Tables 3 and 5). The other RCT study evaluated wounds with impaired wound healing at day 50 and found no difference between no treatment and PRP gel suggesting that PRP therapy does not impede wound healing.^26^ In all studies, PRP therapy had statistically fewer wound-healing disturbances and wound postoperative complications than the no treatment control counterparts^26^^,^^31^^,^^34^ (Table 3).

Six different infection sites and postoperative complications were considered especially important criteria and were addressed in 1 RCT and 4 comparative studies.^25^^,^^31^^,^^33^^-^35 Two studies found infection rates that were not significantly different compared to no topical treatment,^25^^,^^33^ however, chest, superficial and deep infections and postoperative complications were significantly lower in PRP-treated study subjects than in no topical treatment controls^25^^,^^31^^,^^33^^-^35 (Tables 3 and 5). Infection was significantly increased in control groups than that in PRP groups in all but 1 study (Tables 3 and 5).

Exudate, drainage, and hematomas were evaluated in 2 RCT and 2 comparative studies.^26^^,^^30^–^31^^,^^33^ In 1 RCT, PRP-treated wounds had significantly fewer large area hematomas than no topical treatment control wounds, thus reducing a potential source for infection.^26^ The amount and presence of drainage was statistically significantly reduced in the PRP-treated wounds in all studies compared to controls of saline spray or no topical treatment^30^^-^31^,^^33^ (see Tables 3 and 5).

Postoperative, general, resting, and active pain, as well as narcotic use was assessed in 5 RCT studies and 1 comparative study.^26^^-^30^,^^32^ Although 4 RCT studies did not show statistical differences in mean postoperative pain, 1 study did^27^ (Tables 3 and 5). Postoperative pain for both leg and chest were significantly reduced for the PRP-treated wounds for 30 days, but not for no topical treatment control wounds^27^ (Table 3). A comparative study also showed intravenous narcotic use was statistically lower in PRP-treated subjects than in no topical treatment controls indicating less pain was present^32^ (Table 3).

#### Acute secondary closure wounds

Complete wound closure was found to be statistically faster in PRP treated wounds compared to Bacitracin or no topical treatment in both a RCT and comparative study^37^^-^38 (Tables 3 and 5). The healing velocity of PRP-treated wounds was significantly greater than Bacitracin control.^38^ In 1 RCT, the mean time PRP-treated wounds required to partially heal in preparation for definitive surgery was not only significant, but almost half of that of Vaseline gauze controls^36^ (Tables 3 and 5). The PRP group healing rates and wound area and volume reductions were statistically significant in all studies^36^^-^38 (Tables 3 and 5). One RCT study assessed VAS pain scores at 3 weeks and found PRP-treated wounds had significantly less pain than Vaseline gauze controls^36^ (Tables 3 and 5). One RCT administered the SF-36 tool at week 3 to evaluate wound treatment effect on patient QoL and showed PRP-treated subjects had significantly better QoL scores than subjects with no topical treatment^37^ (Table 3).

### Meta-analysis

**Figure 2:**
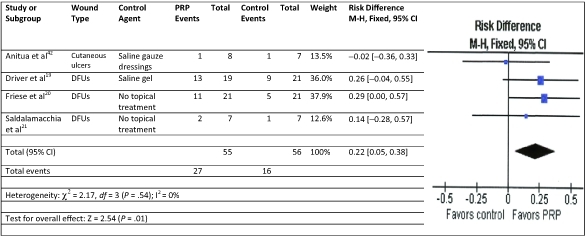
Meta-analysis: Forest plot of chronic wound complete wound healing studies treated with standard wound care and either PRP or control agent. This analysis used the least conservative PP analysis for Driver et al.^19^ CI indicates confidence interval; *df*, degrees of freedom; M-H, Mantel-Haenszel; PRP, platelet-rich plasma.

**Figure 3:**
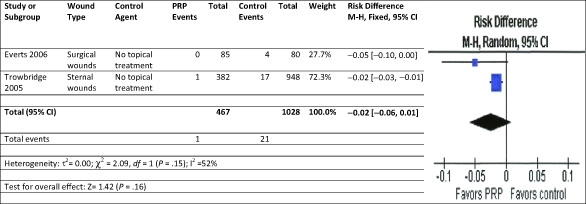
Meta-analysis: Forest plot of acute wounds with primary closure for superficial infection treated with standard wound care and either PRP or control agent. CI indicates confidence interval; *df*, degrees of freedom; M-H, Mantel-Haenszel; PRP, platelet-rich plasma.

**Figure 4:**
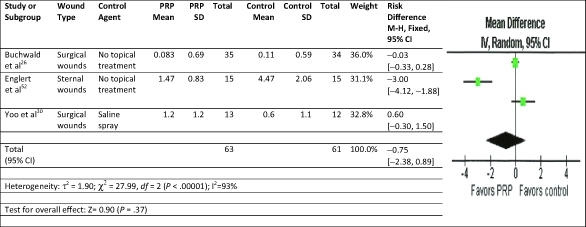
Meta-analysis: Forest plot of acute wounds with primary closure for pain reduction treated with standard wound care and either PRP or control agent. CI indicates confidence interval; *df*, degrees of freedom; M-H, Mantel-Haenszel; PRP, platelet-rich.

A meta-analysis was performed on chronic wound RCTs studies using PRP and standard wound care to analyze the impact of the therapies on complete wound healing.^18^^-^21 Using the fixed-effect model for complete wound healing, the results were significantly in favor of the PRP therapy with no significant heterogeneity compared to control therapies of saline gauze, saline gel, or no topical treatment (Fig 2). A meta-analysis for RCTs in acute wounds with primary closure was not undertaken because there were only 2 studies and their defined outcomes for complete closure were incompatible. A meta-analysis of infection and for pain reduction, however, could be performed. Acute wounds with primary closure comparative studies evaluating superficial infection were modeled using random effects.^31^^,^^35^ The results were in favor of the PRP therapy to reduce infection but not significant compared to no topical treatment (Fig 3). For the acute wound with primary closure, RCT studies evaluating postoperative pain were modeled using random effects.^26^^-^27^,^^30^ The results were in favor of the PRP therapy to reduce pain but not significant compared to saline spray or no topical treatment (Fig 4).

## DISCUSSION

There were 21 publications that were RCT or comparative non-RCT designs in this systematic review. Given the physiological and healing differences between acute and chronic wounds, the citations were divided by study design and by type of wound prior to review and meta-analysis. The primary outcome assessed in this systematic review was complete healing. In both chronic and acute wound studies, complete wound closure was more likely in wounds treated with PRP therapy. Similar partial healing and wound area/volume reduction outcomes were noted more frequently with the PRP-treated wounds likely because the therapy is discontinued once the wound begins healing. This meta-analysis and other systematic reviews show PRP has sufficient efficacy to stimulate healing in stalled wounds. One systematic review concluded that the percentage of total healing in PRP-treated skin ulcers consistently increased compared to controls.^7^ The meta-analysis of chronic wound studies confirmed the use of PRP treatment favored complete healing compared to control care. Other systematic reviews on PRP therapy reached the same conclusions.^7^^-^9 One systematic review went so far as to conclude that based on the meta-analysis and scientific evidence regarding consistent favorable outcomes, PRP is a treatment of choice for the topical care of wounds.^9^

Because nonhealing wounds incur expenses as well as run a higher risk of complications, the sooner a wound can heal or be ready for surgery, the less likely the wound will become more problematic.^83^ The data suggest that PRP therapy can be extremely cost-effective in chronic wounds and it may also be effective in certain acute wounds. A recent study found that a specific PRP gel was the most cost-effective over a 5-year period of time compared to other advanced wound therapies in the treatment of diabetic foot ulcers.^84^ From a clinical point of view, the decrease in complication risks and expenses with healing impacts patient health, finances, and QoL. From a payor's perspective, achieving closure at 25% to 50% faster rate than control care treatment alleviates the expenses of a longer and more problematic treatment course resulting in a benefit for both patient and payor.

Infection and exudate were evaluated in many acute primary closure wound studies because both can delay healing. Overall, infection rates appear to be lower in PRP-treated subjects. The meta-analysis of the RCT studies confirmed the infection rates were lower when PRP treatment was used compared to control care although not significantly so. Because increased exudate in wounds can be a precursor to infection, the presence of the exudate is closely documented. As seen in this review, in both RCT and non-RCT comparative studies, the amount and presence of drainage was statistically significantly reduced in the PRP-treated wounds suggesting potentially fewer infectious complications. Decreasing exudate and infection risks are beneficial for the patient, the wound, and the clinician and should improve the healing rate. Obvious benefits of no or reduced topical and systemic antimicrobials are limiting exposure to microorganisms, reduction in expenses and improved patient QoL. Payors know that wounds that are infected or heavily colonized do not heal as quickly as wounds that are not contaminated, saving costs and time.^2^

Pain is often associated with wounds, but because of the subjective nature of the sensation, determining the impact of wound care therapies on different types of pain, levels of pain, and when pain is assessed can be difficult. Pain outcomes appeared mixed with some studies reporting no difference between treatments and others showing significantly statistical differences favoring PRP treatment. Objective use of measured intravenous narcotic use and SF-36 tool for QoL more clearly demonstrate PRP-treated subjects had significantly better QoL scores and fewer required narcotic medication. This suggests the therapy may result in less wound pain. The meta-analysis suggests PRP therapy may positively impact the patient by reducing pain. Presence of persistent pain can impair healing as well as affect patient QoL.^85^ Reduction in pain allows clinicians to prescribe less addictive and lower risk analgesics as well as fewer drug interactions. While payors sometime underestimate the impact pain has on healing and QoL, the expense of high level pain medication adds up quickly.

The quality of the eligible studies varied widely as shown through multiple scoring methods. Using several methods together allowed the authors to quickly and consistently evaluate the strengths and weaknesses of each paper. Many RCTs were lower in quality, higher in bias, and had more serious limitations than many comparative or cohort studies. This emphasizes the need to evaluate the quality and bias of published studies in addition to the study design.

One limitation of this systematic review was that while many citations evaluated the impact of PRP treatment on wound healing, there are many methods and definitions for determining and measuring wound healing. As the literature pool grows, more subcategories (ie, by PRP product type) can be assessed.

On the basis of the last 10 years of research, the results of this systematic review and meta-analysis suggest that PRP therapy can positively impact wound healing and associated factors such as pain and infection in both chronic and acute cutaneous wounds.

## Acknowledgments

The authors wish to thank their colleagues for their expert opinions in seeking out available studies for the systematic review. The funding for the systematic review was provided by Cytomedix, Inc (Gaithersburg, MD). Carelyn Fylling is an employee of Cytomedix, Inc, and publication of this review was not required as part of her duties. Marissa J. Carter and Laura K. S. Parnell are independent research consultants.

## Figures and Tables

**Table 1:** Description of studies: Types of wounds and interventions used. Intervention group received all care described for control group unless otherwise stated*

Study	Design	N	Study Period	Wound Type	Control Group	Intervention Group
Almdahl et al^25^	RCT	140	6 wk	Leg wounds from long saphenous vein harvesting (CABG)	No topical treatment. Standard closure (intracutaneous poliglecaprone)	Autologous PRP (GPS, Biomet Biologics; activated with autologous thrombin) sprayed prior to closure
Anitua et al^18^	RCT	15	8 wk	Cutaneous ulcers <12cm diameter, ≥4-wk old	Moist saline gauze dressings and cleaning with normal saline; debridement and systemic antibiotics for infection	Autologous PRP (PRGF System, BTI Biotechnology Institute, Vitoria-Gasteiz, Spain) injected once in wound margins.
Buchwald et al^26^	RCT	70	50 d	Leg wounds from long saphenous vein harvesting (CABG)	No topical treatment. Standard closure	Autologous PRP (Angel; Dideco, Mirandola, Italy; activated with autologous thrombin) sprayed prior to closure
Carter et al^22^	Comparative (run-in vs treatment period)	46	≤86 d (run-in); ≤36 d (treatment)	DFUs, PUs, VUs, surgical, dehisced, & traumatic wounds, other types	Run-in period represented control group; authors state variety of moist wound care dressings, dressing changes, debridement as required; compression or offloading per wound type; NPWT for some wounds	Autologous PRP gel treatment (AutoloGel, Cytomedix, Gaithersburg, MD, bovine thrombin) applied to wound bed at least once.
Driver et al^19^	RCT	72	12 wk	DFUs, 1A (U Texas), 0.5-20 cm^2^, ≥4-wk old	Cleaning, dressing changes, debridement as required; offloading; saline gel (Mölynycke Health Care, Norcross, GA) and foam dressing applied after wound bed preparation biweekly for 12 wk or until healed	Autologous PRP gel (AutoloGel, Cytomedix, Gaithersburg, MD, bovine thrombin) applied after wound bed preparation biweekly for 12 wk or until healed
Englert et al^27^	RCT	30	∼30 d	Sternal wounds (CABG)	No topical treatment. Control wound care not reported	Autologous PRP (Magellan, Minneapolis, MN) “caulking bead” applied to sternum with cannula prior to closure
Everts et al^31^	Prospective cohort (controls are consecutive patients who followed)	165	∼1 wk	Surgical wounds (TKA)	Wound drain, no topical treatment, wound dressings not specified, compression bandage	Autologous PRP (Electa, Sorin Group, Mirandola, Italy; 85% activated with autologous thrombin, remaining activated with bovine thrombin) sprayed in back of knee cavity, posterior recess, gutters, etc.) and after deep closure injected on repaired extensor mechanism/prepatellar fat (no wound drain)
Friese et al^20^	RCT	42	25 wk (12 wk for CWH)	DFUs, Wagner 1-3, >0.7 cm^2^, >6-wk old	Cleansing, debridement, dressing changes as needed & offloading. No topical treatment, wound dressed with polyurethane foam	Autologous PRP (Harvest Technologies, Plymouth, MA) every 2 wk for 12 wk
Gardner et al^32^	Retrospective comparison 61 PRP-treated wounds, 37 controls over same time period	98	∼1 wk	Surgical wounds (TKA)	No topical treatment, standard layered closure, dressings used but not specified and use of passive motion device after 24 h	Autologous PRP (Medtronic Sequestra 1000 Autotransfusion System, Medtronic, Minneapolis, MN) injected into posterior recess, gutters, exposed femur/tibia surfaces, repaired extensor mechanism/prepatellar fat (no wound drain)
Hom et al^38^	Prospective comparison of treated wounds with contemporary own patient controls	8 patients, 80 wounds	6 mo	PRP-treated skin punch wounds	Bacitracin topical treatment and semiocclusive dressing	AutoloGel PRP gel (Magellan, Medtronic, Minneapolis, MN; autologous thrombin-rich serum) plus white petrolatum ointment applied once or twice
Kazakos et al^36^	RCT	59	3 wk	Traumatic wounds	Cleansing, debridement, and Vaseline gauze dressings	Autologous PRP gel (PRP Fast system, Bioteck; bench centrifuge; autologous thrombin) applied before or after debridement, and then weekly. Gauze sponges applied following PRP gel.
Khalafi et al^33^	Retrospective analysis with propensity scoring (PRP/controls)	1,128	∼1 wk	Sternal and leg wounds (CABG)	No topical treatment. Control wound care not reported	Autologous PRP (GPS II, Biomet, Inc., Warsaw, IN; activated with bovine thrombin) sprayed into sternal edges/subcutaneous tissue & graft harvest site
Margolis et al^23^	Retrospective cohort study with propensity scoring (PR/controls)	26,599	32 wk	DFUs (neuropathic)	No topical treatment. Standard treatment (moist wound care-not specified, debridement, offloading)	Autologous Platelet Releasate (Curative Health Services, Hauppauge, NY) initiated within the first 12 wk of care
Mazzucco et al^24^	Prospective cohort with historical controls (dehiscent); cohort and controls (ulcers)	2231	1 y	Dehiscent sternal wounds (CABG); necrotic skin ulcers	Daily topical washing/cleaning, and antibiotic therapy as needed (dehiscent wounds); cleaning/dressing with hyaluronic acid/synthetic collagen gauze (ulcers)	Autologous PRP gel (ACD-A Vacutainer tubes, Becton Dickinson Labware, Franklin Lakes, NJ, and bench centrifuge; autologous thrombin) twice per week (dehiscent wounds) or once per week (ulcers) until healed. Covered with Vaseline gauze.
Peerbooms et al^28^	RCT	102	3 mo	Surgical wounds (TKA)	No topical treatment, closed with staples. Wound care dressings not specified. Compression bandages and rehabilitation	Autologous PRP (GPS, Biomet, Inc., Warsaw, IN) sprayed into knee cavity (synovium + cut edges of femur/tibia) and PPP sprayed into subcutaneous tissues; autologous thrombin
Saldalamacchia et al^21^	RCT	14	5 wk	DFUs Wagner 2/3 & ≥8-wk old	No topical treatment, nonspecific standard care	Autologous PRP gel application topically for 5 wk, each week.
Saratzis et al^34^	Comparison 50 prospectively treated PRP-treated wounds with 50 controls over same time period	100	∼30 d	Surgical wounds (inguinal)	No topical treatment, layered closure with sutures and staples. Wound care dressings not specified. Antibiotics, aspirin, clopidogrel, ambulation, and documentation of endograft integrity	Autologous PRP (Magellan, Minneapolis, MN; not activated) injected subcutaneously and percutaneously
Spyridakis et al^37^	RCT	52	30 d	Surgical wounds (pilonidal disease)	No topical treatment, wound care dressings not specified.	Autologous PRP (GPS II system, Biomet, Inc., Warsaw, IN; autologous thrombin) applied into the wound intra-operatively and before postoperatively day 4 and 12
Trowbridge et al^35^	Retrospective comparison PRP-treated wounds with contemporary & historical controls	2,259	Not reported	Sternal wounds (cardiac surgery)	Two control groups both had no topical treatment and standard care that was not specified. One control was a historic control; the other control was performed at time of study.	Autologous PRP (CATS, Terumo Cardiovascular, Ann Arbor, MI; Harvest Technologies, Plymouth, MA; Angel, COBE Cardiovascular, Arvada, CO; bovine thrombin) sprayed to subcutaneous areas, as well as topical application
Vang et al^29^	RCT	38	∼3 wk	Sternal wounds (CABG)	No topical treatment, wound care dressings not specified.	Autologous PRP (Magellan, Minneapolis, MN; bovine thrombin) sprayed into deep tissue and subcutaneous layers
Yoo et al^30^	RCT	52	∼1 wk	Surgical wound (thyroid)	Saline spray used instead of PRP (wound bed) and PPP (under skin incision); Penrose drain (5-min duration) and closed suction drain applied to surgical site after closure.	Autologous PRP (GPS, Biomet, Inc., Warsaw, IN, autologous thrombin) sprayed into wound bed and PPP sprayed under skin incision

*CABG indicates coronary artery bypass graft; CWH, complete wound healing; DFU, diabetic foot ulcer; NPWT, negative pressure wound therapy; PU, pressure ulcer; PPP, platelet-poor plasma; PRP, platelet-rich plasma; PU, pressure ulcer; RCT, randomized controlled trial; TKA, total knee arthroplasty; VU, venous ulcer.

**Table 2:** Quality review of studies: Score sheet. SIGN grade was estimated using the general methodology of Harbour and Miller, assigning a grade based on the total score of external validity, internal validity (bias and confounding) as follows: 0-8 (-); 9-12 (+); 13-16 (++)

	Downs and Black, Carter, Carter							
Study Quality Assessed	Reporting	External Validity	Internal Validity (Bias)	Internal Validity (Confounding)	Power	Score (of 29)	Harbour and Miller SIGN	Author Comments Reason for Upgrade or Downgrade
Anitua et al^18^	9	1	3	3	0	16	−	
Driver et al^19^	10	2	6	1	1	20	−	Downgrade - Efficacy analysis: dropouts high; many treatment violations
Friese et al^20^								*
Saldalamacchia et al^21^								*
Carter et al^22^	8	3	5	3	1	20	+	
Mazzucco et al^24^	10	3	5	2	2	22	+	
Margolis et al^23^	8	3	3	4	0	18	+	
Almdahl et al^25^	11	3	7	5	1	27	++	
Buchwald et al^26^	9	0	7	2	0	18	−	Downgrade- Not clear what treatment controls got
Englert et al^27^	7	3	5	3	0	18	−	Downgrade- Not clear what treatment controls got
Peerbooms et al^28^	9	2	7	3	1	22	++	Upgrade- ITT analysis showed better results than PP
Vang et al^29^	10	2	4	1	2	19	−	
Yoo et al^30^	9	2	6	5	0	22	++	
Everts et al^31^	8	3	5	2	0	18	+	
Gardner et al^32^	5	3	4	2	0	14	+	
Khalafi et al^33^	7	3	3	2	0	15	+	Upgrade- Large N, propensity scoring techniques used
Saratzis et al^34^	10	2	6	4	0	22	+	
Trowbridge et al^35^	9	3	4	4	0	20	+	
Kazakos et al^36^	10	3	5	2	0	20	+	
Spyridakis et al^37^	8	3	5	3	0	19	+	
Hom et al^38^	10	2	4	2	0	18	−	

*Friese citation was a RCT abstract and the Saldalamacchia citation was a research letter. Neither of these two citations could be scored. Both citations are included because Cochrane would have included them in their analysis.

**Table 3:** Detailed outcomes reported for the study period. Number needed to treat (NNT) was calculated based on complete wound healing information provided in the publication*

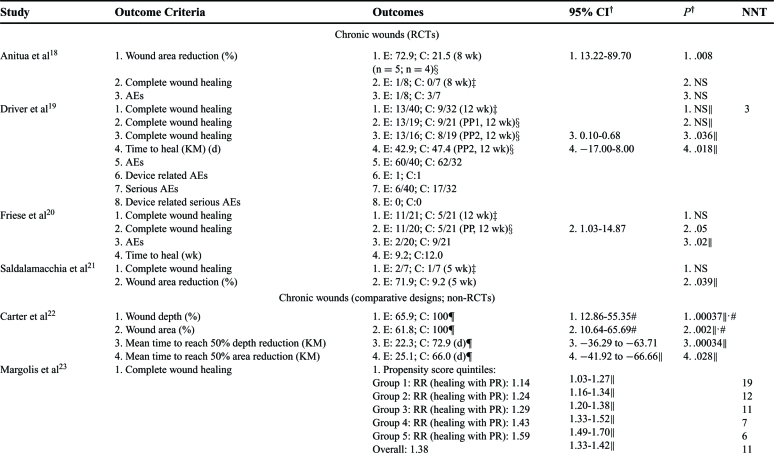
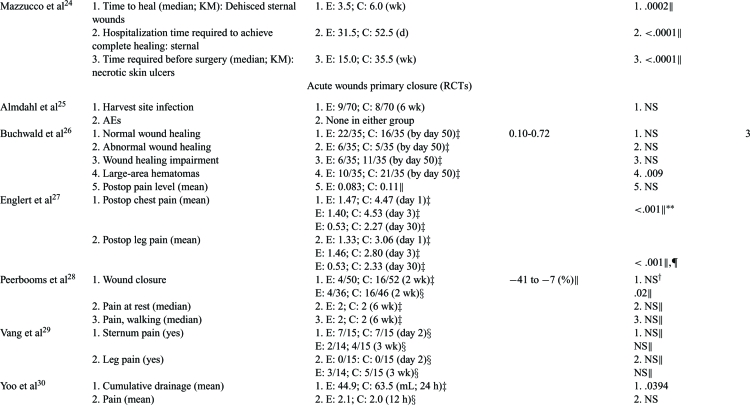
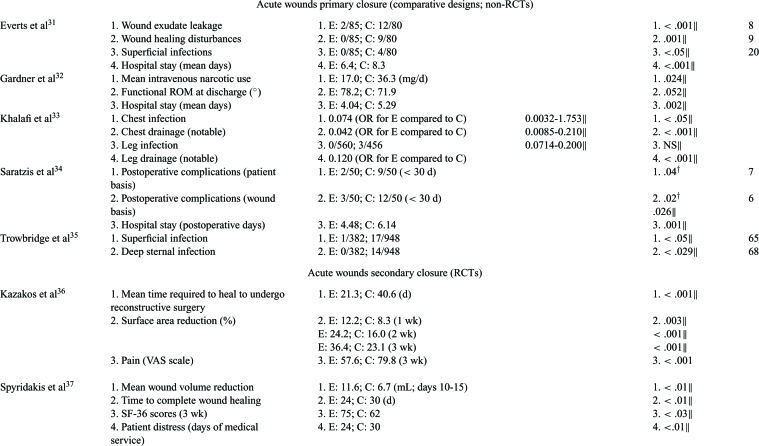
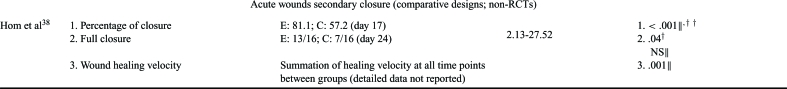

*Wound size reductions are reported as mean reductions unless otherwise stated; CIs are calculated for risk difference in dichotomous outcomes and for weighted mean difference in continuous outcomes unless otherwise stated. AE indicates adverse event; C, control group (comparison); CI, confidence intervals; E, experimental group (PRP); KM, Kaplan-Meier; NNT, number needed to treat (based on complete wound healing); NS, not significant; OR, odds ratio; PRP, platelet rich plasma; PR, platelet releasate; RR, relative risk.

^†^ Values calculated using the *Z* test (fixed effects) when significant unless otherwise indicated (ie, author values).

‡Intention-to-treat (ITT) analysis.

§ Per protocol (PP) analysis.

∥Author values.

¶C represents wounds during a run-in period and E represents same wounds during treatment period.

# Multivariate repeated measures general linear model in which 95% CI is for mean difference; values for C represent area or depth at first pretreatment value and for E represent percent area or depth at last treatment time.

** Repeated-measures ANOVA: multivariate for chest pain (Wilk's λ = 0.43) and univariate for leg pain.

†† Repeated-measures ANOVA, 42 days.

**Table 4:** Quality assessment and summary of findings for studies comparing use of platelet-rich plasma treatments against standard care for chronic wounds

Quality Assessment						Summary of Findings					
						No of Patients		Effect			
No of Studies	Design	Quality	Consistency	Directness	Other Modifying Factors	PRP	Controls	Relative (95% CI)	Absolute	Quality	Importance
Complete Wound healing											
4	RCT	Serious limitations	Some inconsistency	No uncertainty	Small trials (power issues)	76	67	RD: 0.24 (0.07-0.40)*	22/100	Low	Critical
1	Comparative	No serious limitations	No inconsistency	No uncertainty	Large N, well-done analysis, evidence of better healing for severe wounds	6,252	20,347	RR: 1.38 (1.33-1.42)	9/100	Mod	Critical
Time to heal (days)											
1	RCT	Serious limitations	Some inconsistency	No uncertainty	Numerous protocol violations	19	21	WMD: -4.50 (–17.0 to 8.0)	−4.5 d	Low	Critical
1	Comparative	No serious limitations	No inconsistency	No uncertainty	Small N	10	12	—	−17.5 d	Low	Critical
Mean time to reach 50% depth or area reduction											
1	Comparative	No serious limitations	No inconsistency	No uncertainty	Relatively small N	41	46	Depth: WMD: −50.6 (–37.56 to −63.64)	Depth: 3.3-fold	Mod	Critical
						39	46	Area: WMD: −40.9 (−26.19 to -55.61)	Area: 2.6-fold		
Adverse events											
3	RCT	Some limitations	No inconsistency	No uncertainty	Small aggregate N	63	74	—	NNH: 11	Low	Important

*Data from one RCT uses intermediate PP results (N = 19/21); mod indicates moderate; NNH, number needed to harm; RCT, randomized controlled trial; RD, risk difference; RR, relative risk; WMD, weighted mean difference.

**Table 5:** Quality assessment and summary of findings for studies comparing use of platelet-rich plasma treatments against standard care for acute wounds*

Quality Assessment						Summary of Findings					
						No of Patients		Effect			
No of Studies	Design	Quality	Consistency	Directness	Other Modifying Factors	PRP	Controls	Relative (95% CI)	Absolute	Quality	Importance
**Acute wounds (primary closure)**											
Complete wound healing											
1	RCT	No limitations	No inconsistency	No uncertainty	Short follow-up	50	52	RD: −0.23 (−0.37 to −0.08)	−23/100	Mod	Critical
Infection											
1	RCT	No limitations	No inconsistency	No uncertainty	Leg infection	70	70	RD: 0.01 (−0.09 to 0.12)	1.4/100	High	Important
1	Comparative	No serious limitations	No inconsistency	No uncertainty	Leg infection; large N	560	546	RD: −0.01 (−0.01 to 0)	−5.5/1000	Mod	Important
2	Comparative	No serious limitations	No inconsistency	No uncertainty	Superficial infection	467	1028	RD: −0.02 (−0.06 to 0.01)	−22/1000	Mod	Important
1	Comparative	No serious limitations	No inconsistency	No uncertainty	Chest infection; large N; propensity scoring	571	557	OR: 0.0743 (0.0032-1.7535)	—	Mod	Important
1	Comparative	No serious limitations	No inconsistency	No uncertainty	Chest infection	382	948	RD: −0.01 (−0.02 to −0.01)	14.8/1000	Mod	Important
Pain reduction											
3	RCT	Some serious limitations	Inconsistency between studies	Some uncertainty (overall vs. chest pain)	Small N	63	61	WMD: −0.75 (−2.38 to 0.89)	−0.75/10	Very low	Important
1	RCT	No serious limitations	No inconsistency	Some uncertainty		50	52	—	No difference	Mod	Important
1	RCT	Serious limitations	No inconsistency	Some uncertainty	Dichotomous outcomes	15	15	RD: 0 (−0.36 to 0.36)	No difference	Very low	Important
Wound drainage/exudate											
1	RCT	No serious limitations	No inconsistency	No uncertainty	Small N	26	26	WMD: 18.6 (36.92 to 0.28)	18.6 mL	Low	Important
1	Comparative	No serious limitations	No inconsistency	No uncertainty	Large N; propensity scoring	571	557	OR: 0.042	—	Mod	Important
1	Comparative	No serious limitations	No inconsistency	Some uncertainty		85	80	RD: −0.13 (−0.21 to −0.04)	−13/100	Mod	Important
**Acute wounds (secondary closure)**											
Complete wound healing											
1	Comparative	Serious limitations	No inconsistency	No uncertainty	Small N	16	16	RD: 0.31 (−0.02 to 0.64)	31/100	Very low	Critical
Time to complete wound healing											
1	RCT	No serious limitations	No inconsistency	No uncertainty	Small N	30	22	—	6 d	Low	Critical
Wound volume reduction											
1	RCT	No serious limitations	No inconsistency	No uncertainty	Small N	30	22	WMD: 4.9 (3.79-6.01)	4.9 mL	Mod	Important
Area reduction (quotient method)											
1	RCT	No serious limitations	No inconsistency	No uncertainty	Small N	32	27	WMD: 0.13 (0.08-0.18)	0.13 cm^2^	Mod	Important
Pain											
1	RCT	No serious limitations	Some inconsistency	No uncertainty	Small N	32	27	WMD: −22.2 (−31.16 to −13.28)	−22.2/100	Low	Important

*Mod indicates moderate; OR, odds ratio; RCT, randomized controlled trial; RD, risk difference; RR, relative risk; WMD, weighted mean difference.
